# Differential expression of miRNAs as biomarkers for predicting the outcomes of diffuse large B-cell lymphoma patients

**DOI:** 10.1042/BSR20201551

**Published:** 2021-06-28

**Authors:** Maogui Hu, Xinchen Wang, Ning Liu, Kaiyang Ding, Guihong Zhang, Xiaosi Liu

**Affiliations:** 1 Department of Hematology, Anhui Provincial Hospital, The First Affiliated Hospital of USTC, Hefei 230001, China; 2 Department of Pathology, The First Affiliated Hospital of Anhui Medical University, Hefei 230032, China

**Keywords:** diffuse large B-cell lymphoma, microarray, miRNA, prognosis, qRT-PCR

## Abstract

**Background:** Diffuse large B-cell lymphoma (DLBCL) used to be defined as germinal center B-like and non-germinal center B-like subtypes, associated with different prognoses, but the conventional classification does not meet the needs of clinical practice because of DLBCL heterogeneity, a problem that might be improved by selection of miRNAs as biomarkers.

**Methods:** Twelve patients with DLBCLs were used to screen out the aberrant miRNA profile using miRNA microarray technology in two patient subtypes (six germinal center B-like and six non-germinal center B-like patients). The potential biomarkers were further analyzed using the quantitative reverse transcription-polymerase chain reaction method in 95 DLBCL patients to investigate relationships among expression levels of potent miRNA, clinicopathological features and survival rates of patients.

**Results:** miR-208a-5p, miR-296-5p and miR-1304-5p were screened as potential biomarkers. miR-208a-5p and miR-296-5p were shown to be associated with better survival of patients after Kaplan–Meier analysis, whereas miR-1304-5p overexpression indicated a poor survival prognosis independent of the DLBCL subtype. In addition, changes of miR-296-5p and miR-1304-5p expression, the International Prognostic Index (IPI) status and the age of patients were all independent indicators for DLBCL prognosis. We also found that high miR-208a-5p expression led to better outcomes in DLBCL patients with similar IPI scores; however high miR-1304-5p expression tended to indicate the opposite.

**Conclusions:** MiR-208a-5p, miR-296-5p and miR-1304-5p levels might be potential biomarkers for the prediction of the prognosis of DLBCL patients.

## Introduction

Diffuse large B-cell lymphoma (DLBCL) is recognized as one of the most prevalent subtypes of non-Hodgkin’s lymphoma [1], which can be further divided into two distinct subtypes, namely the germinal center B-like DLBCL (GCB-DLBCL) and the non-germinal center B-like subtype (non-GCB) according to the Hans’ system [2,3]. The non-GCB phenotype is associated with substantially worse outcomes when treated with standard chemoimmunotherapy, whereas GCB has a better 5-year survival rate than for non-GCB subtype patients [1,2]. To date, treatment of DLBCL has consisted of the administration of rituximab, cyclophosphamide, doxorubicin, vincristine and prednisone (R-CHOP), leading to a complete remission rate of approximately 75% [4]. Nevertheless, even with R-CHOP-like treatment regimes, 30–40% of patients ultimately suffer from tumor relapse or progression [5]. Most patients survive for a median time of 1 year, with almost 50% suffering a relapse after 1 year [6]. Thus, there is an urgent need to find a novel way to identify poor outcome DLBCL cases and to elevate the survival time by prescribing optimal individualized therapy.

During the past decades, there have been a number of scientific breakthroughs that have provided important insights into DLBCL biology, such as the exploration of gene expression profiles to estimate outcomes [7–9], predicting the subtypes of DLBCL by using immunohistochemistry-based algorithms [10], as well as carrying out exome and transcriptome sequencing to identify genetic drivers of the disease [11]. However, gene expression profiles are impractical in routine clinical practice due to the high cost and routine immunohistochemistry studies that measure aberrant protein expression in tumor specimens may not be sufficient for DLBCL subtype identification [12,13]. There are also some inconsistencies between immunohistochemical and gene expression profiling studies, which result in obstructions for DLBCL prognoses prediction [14]. In medical practice, the clinic-based International Prognostic Index (IPI) is often used to stratify DLBCL patients for treatment selection, but it provides only an insufficient understanding of the mechanisms driving this disease and has limited predictive powers [5,15].

miRNAs are small endogenous, single-stranded RNAs that are non-coding and play intimate but vital roles in gene expression post-transcriptionally, either by inhibiting mRNA translation or by activating the degradation of mRNA [5]. When miRNAs are expressed aberrantly, they have profound effects on lymphocyte biology and play important roles in the mechanisms promoting the development of DLBCL [16]. Recently, miRNAs in the circulation have been suggested to be potential biomarkers for various forms of these cancers [17]. miR-155 was the first used in lymphoma research and is thought to be associated with NF-κB [18] and is an unfavorable factor for the prognosis of DLBCL patients. In addition, TP53 is thought to be involved in the development of DLBCL by direct regulation of HSA-miR-34a-5p acting through p53 signaling pathways [19].

In the present study, we first profiled differentially expressed miRNAs between GCB-DLBCL and non-GCB-DLBCL subtypes using an miRNA microarray. Three miRNAs were screened out and utilized as potential biomarkers to compare the altered expression in the validation cohort of DLBCL patients. The clinicopathological parameters and survival rates were measured to investigate the correlation between miRNA expression and DLBCL patient outcomes. We tried to provide insightful information for the aberrant expression of three miRNAs as biomarkers that can potentially be used to predict the likely course of DLBCL.

## Methods

### Patient specimens

Twelve tissue specimens were first fixed with formalin and then embedded in paraffin (FFPE), taken from six GCB and six non-GCB subtype DLBCL patients. They were first used to determine the miRNA microarray profiling sequences and for quantitative reverse transcription-polymerase chain reaction (qRT-PCR) validation analysis. Ninety-five DLBCL patients (41 cases of GCB, 54 cases of non-GCB) were then retrospectively enrolled for further validation of differential miRNA expression by qRT-PCR. Specimens from eight patients with reactive lymphoid hyperplasia served as the controls for lymphoma verification in the miRNA microarray screening, since they have distinctly different miRNA pattern compared with lymphoma [20]. The DLBCL patients were first diagnosed with DLBCL using the WHO (2016) tumor classification guidelines for the hematopoietic system [21]. Subtypes were characterized immunohistochemically using monoclonal antibodies against MUM1, BCL6 and CD10, based on Hans’ system [2]. From 2006 to 2011 in the First Affiliated Hospital of Anhui Medical University, all specimens were acquired on first diagnosis of the disease from the Department of Pathology but before any treatment commenced. The patients were given uniform R-CHOP therapy in the Department of Hematology. Clinical data including gender, age, serum lactate dehydrogenase (LDH) levels, tumor location and size, Eastern Cooperative Oncology Group (ECOG) performance status, stage and IPI scores were extracted from the medical records of patients. The IPI scores were evaluated according to the following risk factors [22]: age ≥ 60 years; elevated LDH level; stage III or IV disease; ECOG performance status ≥ 2 and 2 or more extranodal sites, with each factor counting as 1 point. Based on the resulting IPI score, patients could be categorized as low risk (0–1 point), low-intermediate risk (2 points), high-intermediate risk (3 points) and high risk (4–5 points) cases. With this model, relapse-free and overall survival (OS) rates at 5 years were estimated as follows: 75% for 0–1 risk factors, 50% for 2–3 risk factors and 25% for 4–5 risk factors. The Ethics Committee of the First Affiliated Hospital and Provincial Hospital Affiliated to Anhui Medical University granted permission for the investigation; all patients signed informed consent forms before being enrolled.

### miRNA microarray analysis

Tissue specimens (*vide supra*) were analyzed by miRNA microarray using a miRCURY LNA™ microRNA array (7th generation, Exiqon, Denmark), based on miRBase version 18.0. Briefly, RNA was isolated using TRIzol® Reagent (Invitrogen, Carlsbad, U.S.A.) and then purified with an RNeasy mini kit (Qiagen, Hilden, Germany). The quantity and quality of the extracted RNA was determined with the aid of an ND-1000 Nanodrop spectrophotometer (Nanodrop Technologies, U.S.A.) and the veracity of RNA was established by using gel electrophoresis.

Each microarray slide was scanned using an Axon GenePix 4000B microarray scanner (Axon Instruments, U.S.A.) and examined using GenePix Pro version 6.0 (Axon) to measure the intensity of each raw image.

### Analysis using the qRT-PCR

A qRT-PCR was carried out to authenticate miRNA expression of the microarray data using a SYBR® Green Two-Step Kit with ROX (Takara, Dalian, China). In particular, RNA was isolated from the tissue specimens (10 × 10 µm sections) with TRIzol® Reagent purchased from Invitrogen (Carlsbad, U.S.A.) and subsequently purified using an RNeasy mini kit (Qiagen, Hilden, Germany). The RNA was then converted into cDNA using specific miRNA primers (Invitrogen) and a PrimeScript™ RT Reagent Kit (Takara, Japan). qPCR was then carried out using SYBR® Premix ExTaq II (Takara, Japan). The reverse transcriptase primers and PCR primers for the miRNAs are shown in Supplementary Table S1. Expression of miRNA was normalized with RNU6 as the calibrator using the 2^−ΔΔ*C*_t_^ method: Δ*C*_t_ = *C*_t_ (miRNA) − *C*_t_ (U6), ΔΔ*C*_t_ = Δ*C*_t_ (case) − mean Δ*C*_t_ (control), with triplicates of each specimen being analyzed to increase the reliability of the data.

### Statistical analysis

During the analysis of microarrays, miRNA expression was normalized with RNU6 to the median level and reactive lymphoid hyperplasia tissue specimens served as non-lymphoma controls for the microarray profiling. Subsequently, differentially expressed miRNAs were assessed for statistically significant differences using Wilcoxon ranked-sum test with multiple test corrections (Benjamini–Hochberg-adjusted *P*-value, with a threshold of false discovery rate (FDR) < 0.05) and a fold change ≥ 2.0 as the cut-off value between non-GCB and GCB subtypes. Any changes in miRNA levels in patients with different clinical characteristics were compared using a *t* test or a chi-squared test as appropriate. To ensure the involved miRNAs were common, any correlations among the three miRNAs were assessed using Pearson’s correlation test. miRNAs with significant negative correlations (FDR < 0.05) were filtered out. Receiver operating characteristic (ROC) curve analysis was performed to define optimal cut-off values of the selected miRNAs on the basis of the subtypes of DLBCL (GCB and non-GCB). Kaplan–Meier analysis with a log-rank test and Cox proportional hazards regression analysis was employed to estimate the survival time based on the IPI status (0–2 or 3–5). A two-sided *P*-value <0.05 was deemed to be a statistically significant finding. All statistical analyses were carried out using SPSS version 16.0 (SPSS, U.S.A.) or GraphPad Prism version 5.0 (GraphPad Software, La Jolla, U.S.A.).

## Results

### Differential expression of miRNAs between GCB and non-GCB subtypes in different specimens and measurement methods

**Figure 1:**
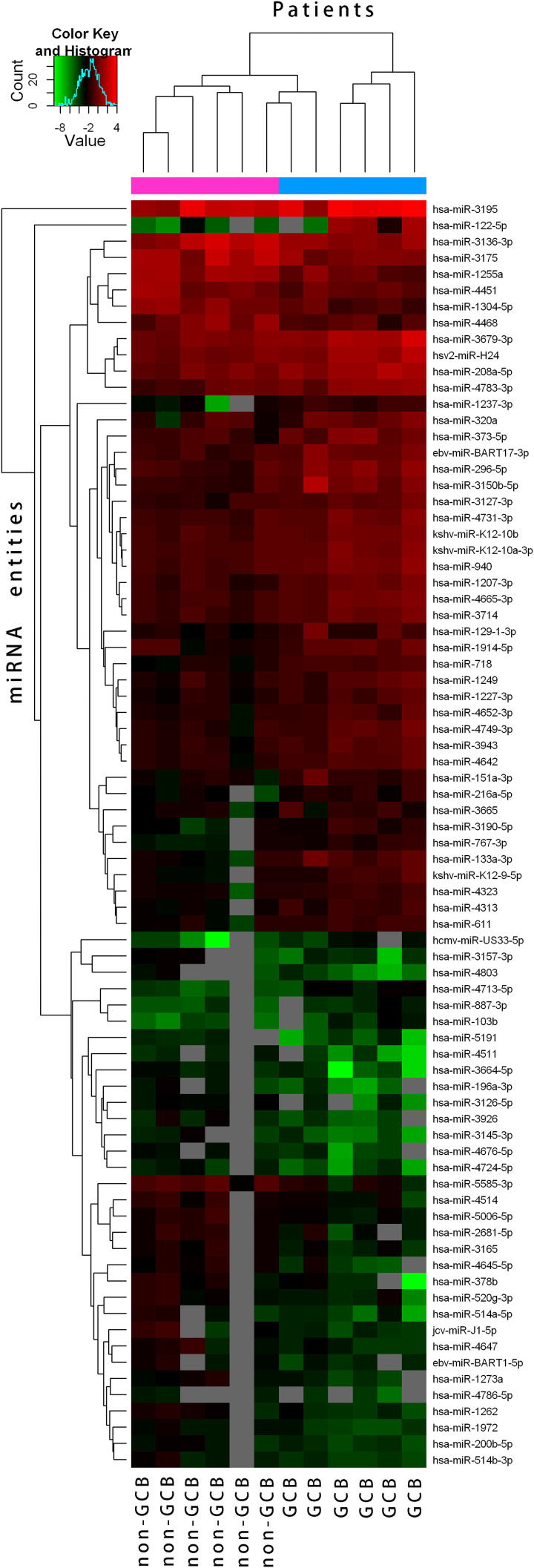
Heatmap from 12 DLBCL samples (6 GCB cases and 6 non-GCB cases) after microarray analysis

**Figure 2:**
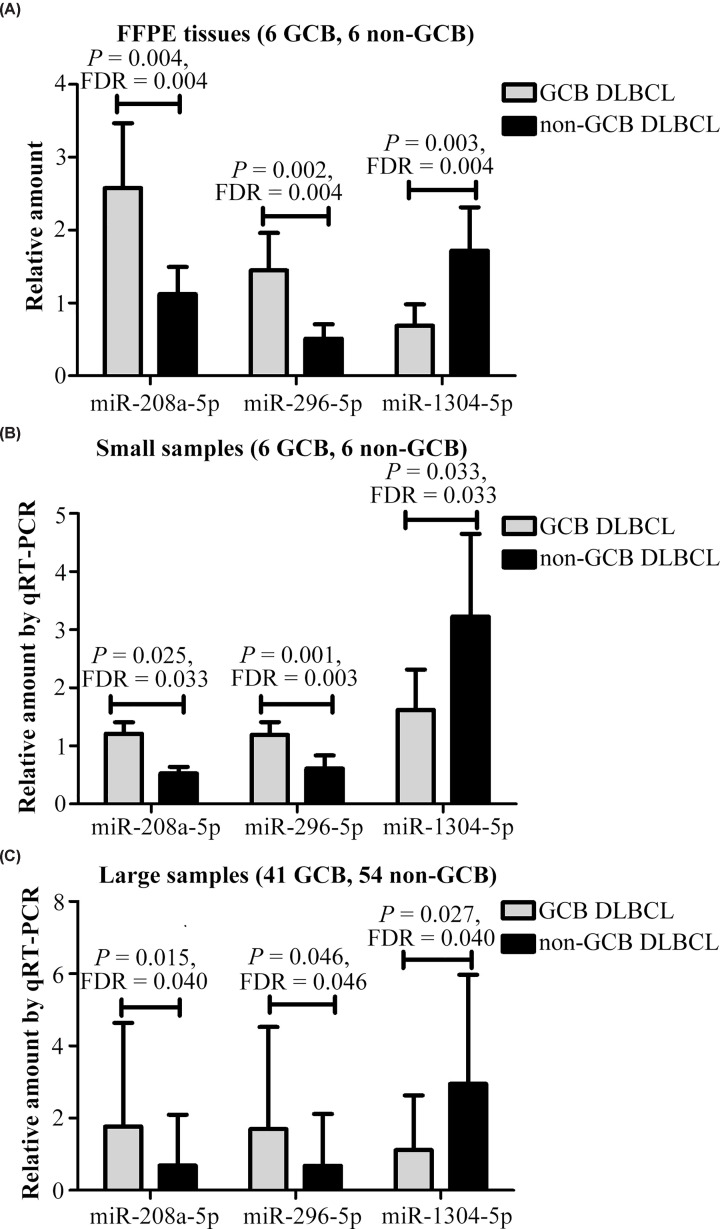
The relative expression levels of the three most differentially expressed miRNAs measured by qRT-PCR in GCB and non-GCB DLBCL specimens (**A**) Analysis after specimens were fixed with formalin and then embedded in paraffin (FFPE). (**B**) Relative expression confirmation of the three miRNAs measured by qRT-PCR in GCB and non-GCB cases. (**C**) Altered miRNAs’ expressions validated in 95 DLBCL specimens by using qRT-PCR.

The miRNA expression patterns from the 12 DLBCL specimens (6 GCB cases and 6 non-GCB cases) after microarray analysis revealed significant difference between GCB and non-GCB (**heatmap** Figure 1); in particular, the three most differentially expressed miRNAs were detected in DLBCL, which were miR-296-5p, miR-208a-5p and miR-1304-5p. Compared with non-GCB DLBCL cases, miR-296-5p and miR-208a-5p levels in GCB were significantly up-regulated, while miR-1304-5p was most highly expressed in non-GCB subtype cases as revealed by microarray analysis (Figure 2A). These three miRNAs expression levels in 12 DLBCL patients were confirmed again by qRT-PCR (Figure 2B). These altered miRNAs were further validated in another 95 DLBCL specimens, which produced similar results using qRT-PCR (Figure 2C). Furthermore, correlation analysis demonstrated that the relative expression of miR-1304-5p was significantly related to miR-208a-5p and miR-296-5p expression (r = 0.278, *P*=0.012 and *r* = 0.378, *P*=0.001, respectively) (Supplementary Table S2 and Figure S1).

### Association of miRNA expression with clinicopathological parameters

To investigate whether the expression of the three miRNAs was correlated with clinicopathological parameters, we categorized the expression in DLBCL specimens as high vs low, based on the mean of each relative miRNA expression level (2^−ΔΔ*C*_t_^). The associations between miRNA levels and clinical parameters are shown in Table 1. Specifically, miR-208a-5p was highly expressed in DLBCL patients and had a significant association with a lower stage (*P*=0.013) and lower IPI scores (*P*=0.001). High miR-296-5p expression tended to occur in patents with lower IPI scores (*P*=0.026), whereas high miR-1304-5p levels were linked to higher IPI scores (*P*=0.020) (Table 1). Comparisons of white blood cell counts, hemoglobin (Hb) levels, platelet counts as well as liver and kidney functions between patients with high and low miR-208a-5p, miR-296-5p and miR-1304-5p expressions revealed that only Hb data were significantly different between high and low miR-208a-5p expression (*P*=0.049) and alanine aminotransferase (ALT) for high and low miR-296-5p expressions (*P*=0.021), but all Hb and ALT values were within the normal ranges (Supplementary Table S3).

**Table 1:** Differentially expressed miRNAs level and clinicopathological data from DLBCL patients

Factors	miR-208a-5p			miR-296-5p			miR-1304-5p		
	High (*n*, %)	Low (*n*, %)	*P*-value	High (*n*, %)	Low (*n*, %)	*P*-value	High (*n*, %)	Low (*n*, %)	*P*-value
**Gender**									
Male	25 (49.0)	26 (51.0)	0.152	16 (31.4)	35 (68.6)	0.822	21 (41.2)	30 (58.8)	1.000
Female	15 (34.1)	29 (65.9)		12 (27.3)	32 (72.7)		17 (38.6)	27 (61.4)	
**Age (years)**									
≥60	21 (42.9)	28 (57.1)	0.878	14 (28.6)	35 (71.4)	0.842	25 (51.0)	24 (49.0)	0.036
<60	19 (41.3)	27 (58.7)		14 (30.4)	32 (69.6)		13 (30.4)	33 (69.6)	
**Extranodal site involvement**									
Yes	17 (47.2)	19 (52.8)	0.430	8 (22.2)	28 (77.8)	0.255	15 (41.7)	21 (58.3)	0.796
No	23 (39.0)	36 (61.0)		20 (33.9)	39 (66.1)		23 (39.0)	36 (61.0)	
**LDH**									
High*	12 (36.4)	21 (63.6)	0.408	6 (18.2)	27 (81.8)	0.100	14 (42.4)	19 (57.6)	0.725
Normal	28 (45.2)	34 (54.8)		22 (35.5)	40 (64.5)		24 (38.7)	38 (61.3)	
**Tumor size**									
≥10 cm	15 (46.9)	17 (53.1)	0.518	5 (15.6)	27 (84.4)	0.056	12 (37.5)	20 (62.5)	0.723
<10 cm	25 (39.7)	38 (60.3)		23 (36.5)	40 (63.5)		26 (41.3)	37 (58.7)	
**ECOG (PS)**									
2–4	22 (39.3)	34 (60.7)	0.505	18 (32.1)	38 (67.9)	0.648	20 (35.7)	36 (64.3)	0.307
0–2	18 (46.2)	21 (53.8)		10 (25.6)	29 (74.4)		18 (46.2)	21 (53.8)	
**Stage**									
III–IV	14 (29.2)	34 (70.8)	0.013	12 (25.0)	36 (75.0)	0.374	23 (39.7)	35 (60.3)	0.932
I–II	26 (55.3)	21 (44.7)		16 (34.0)	31 (66.0)		15 (40.5)	22 (59.5)	
**IPI**									
3–5 scores	14 (26.4)	39 (73.6)	0.001	11 (20.4)	43 (79.6)	0.026	27 (50.9)	26 (49.1)	0.020
0–2 scores	26 (61.9)	16 (38.1)		17 (41.5)	24 (58.5)		11 (26.2)	31 (73.8)	

* Standard of high LDH is defined as >250 IU/l.

### Association of miRNA expression with prognosis of DLBCL patients and their IPI scores

**Figure 3:**
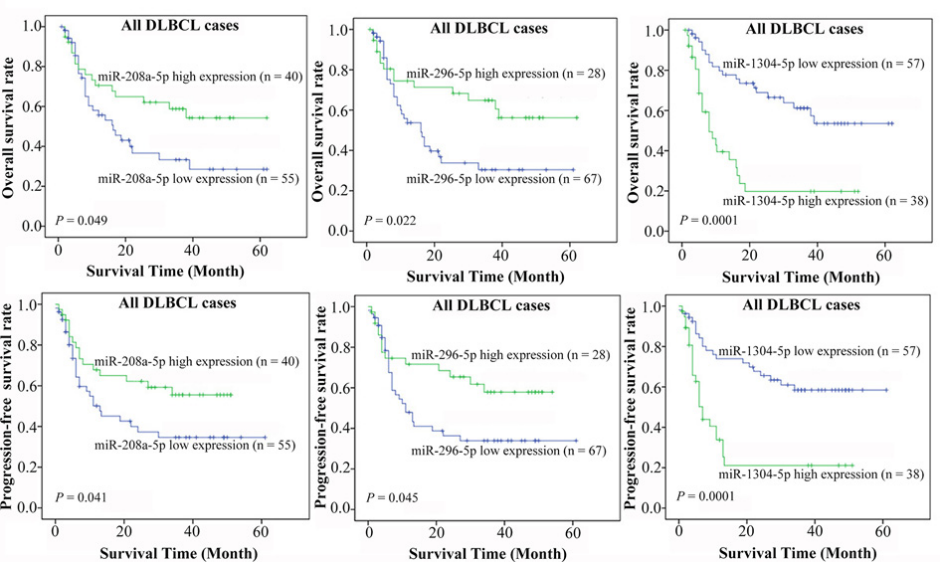
Association of miR-208a-5p and miR-296-5p as well as miR-1304-5p expression and OS or PFS rates among the 95 DLBCL patients using the Kaplan–Meier analysis method

Correlations between overall and progression-free survival (PFS), and miRNA expression in DLBCL patients were also analyzed. The results of Kaplan–Meier analysis showed that higher miR-208a-5p and miR-296-5p expression correlated with better overall and PFS among the 95 DLBCL patients. In contrast, high expression of miR-1304-5p was associated with both reduced OS and PFS (Figure 3).

**Figure 4:**
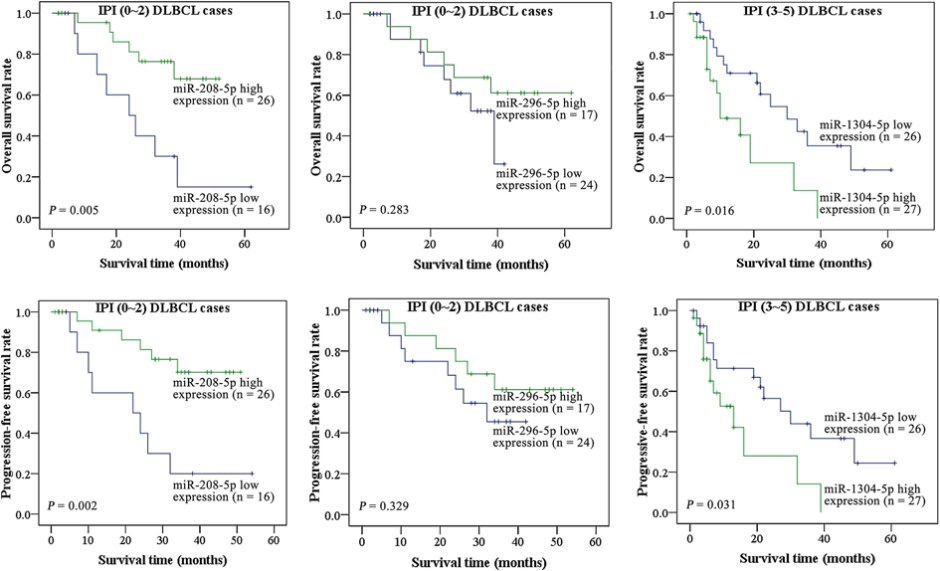
Kaplan–Meier curves after stratification of correlations of miRNAs expression and prognostic outcomes in subtypes of IPI (0–2) and IPI (3–5) DLBCL cases

Moreover, we also investigated whether these miRNAs could provide further prognostic information among patients with similar IPI scores. A total of 95 DLBCL patients were classified into two subgroups according to their IPI scores. In the IPI (0–2) group, patients with higher expression of miR-208a-5p had better OS and PFS rates, but OS and PFS were worse in patients with higher expression of miR-1304-5p in the IPI (3–5) group (Figure 4).

The results of multivariate analyses for OS are summarized in Table 2. According to the univariate analysis, age, IPI score, miR-208a-5p, miR-296-5p and miR-1304-5p were associated with the outcomes of DLBCL patients. Multivariate analysis revealed that age, IPI scores, miR-296-5p and miR-1304-5p were independent indicators for DLBCL prognoses (Table 2).

**Table 2:** Multivariate analysis of the OS of lymphoma patients

Variable	*P*-value	HR	95% CI
Age (>61 vs ≤60 years)	0.003	4.121	1.622–10.471
IPI status (3–5 vs 0–2 scores)	0.033	1.243	1.066–1.890
miR-208a-5p (high vs low)	0.249	0.628	0.285–1.385
miR-296-5p (high vs low)	0.020	0.398	0.184–0.863
miR-1304-5p (low vs high)	0.001	5.259	2.364–11.700

## Discussion

B-cell lymphomas with different genetic abnormalities are a heterogeneous group of DLBCLs, with differing clinical symptoms, responses to therapy and prognoses [12]. Research in past decades has provided interesting insights into the pathogenesis of DLBCL and associated molecular alterations. Accumulating data suggest that miRNAs are promising as ideal diagnostic or prognostic biomarkers, based on cell lines or tumor tissue specimens [23]. Only a small number of miRNAs showed significant potential in more than one study. Therefore, more clinical validation including prospective and cross-center studies are required before specific miRNAs can be integrated into daily clinical practice as biomarkers for DLBCL, which reflects the dawn of the era of more personalized medicine [24]. For example, the expression of miR-21, miR-155 and miR-221 has been found to be up-regulated in the non-GCB subtype compared with the GCB subtype of cancer [25]. Additionally, up-regulation of miR-221-3p and down-regulation of let-7c-5p in the non-GCB type, and up-regulation of miR-363-3p and down-regulation of 423-5p in patients with GC-DLBCL, has recently been reported [26]. Yang et al. also demonstrated a role for miR-197 as a biomarker with potential therapeutic implications [27]. In the current study, three aberrantly expressed miRNAs were screened out by miRNA microarray analysis, which revealed a significant association between patient DLBCL subtypes and IPI scores. miR-208-5p was considered to promote cell metastasis and invasion in a pancreatic cell line through the AKT/GSK-3β/snail signaling pathway [28]. Previous studies have suggested that miR-296-5p is a tumor suppressor in lung, prostate and various other types of cancer. It was also thought to be linked to poor chemotherapeutic efficacy in gastrointestinal tumors, which indicated the heterogeneity of miR-296-5p in different tissues [29–31]. In gastric cancer, miR-1304-5p participates in the circ-PRMT5/miR-145/miR-1304/MYC axis to promote tumor progression and the MYC gene is also associated with ‘double hit’ DLBCL, which indicates an unfavorable prognosis [32]. Multivariate analysis was carried out in the present study and demonstrated that altered miR-296-5p and miR-1304-5p expression, IPI status and patient age were all independent indicators for prognosis. Additionally, we showed that miR-208a-5p and miR-1304-5p also exhibited good prognostic value in patients with similar IPI scores. These findings imply that the detection of these three miRNAs could be useful biomarkers for screening in order to identify the potential risk of DLBCL, which is critical for the selection of a treatment regimen.

miRNA, as an indicator of tumor diagnosis sensitivity and specificity, could also be directly associated with disease progression or treatment outcomes [33]. For example, miR-21 was reported to have potential to serve as a biomarker for genotyping, early diagnosis and prognosis, as well as for treatment options in Chinese DLBCL patients [34]. In addition, miR-18a expression was shown to be associated with the OS time. In contrast, miR-181a and miR-222 expression was associated with PFS, determined in 176 DLBCL patients treated in a uniform manner [35]. The specific miRNA expression patterns were highly associated with the outcomes of DLBCL patients. Our data indicated that up-regulation of miR-208a-5p and miR-296-5p, and down-regulation of miR-1304-5p was associated with both better OS and PFS of patients with DLBCL. Altered expression of any of the three miRNAs studied may contribute to the development of DLBCL and its progression by functioning as tumor suppressor genes or playing an oncogenic role. Thus, the detection of these three miRNAs together may well be a useful biomarker for the prognostic evaluation of DLBCL patients.

Although the predictive effect of miRNAs in treating DLBCL patients has been widely studied, the underlying mechanisms driving disease progression remain to be unequivocally established. Montgomery et al. [36] demonstrated that miR-208a may serve as a biomarker during heart disease progression [37]. It was also shown previously that miR-296-5p could act to suppress tumor growth, with the capability of inhibiting malignant transformations and progression by targeting Pin1, for example in prostate cancer [38]. Gene target prediction analysis suggested that miR-208a-5p and miR-296-5p have the same targets as SMAD4 and SMAD2 in the WNT signaling pathways (http://www.targetscan.org). In another study, it was shown that overexpression of hsa-miR-1304 was associated with the apoptotic characteristics of Bcl-xL in human lung adenocarcinoma cells, which had been silenced by linking them to the WNT signaling pathway [39]. To our knowledge, this is the first report of altered miR-296-5p, miR-208a-5p and miR-1304-5p expression in DLBCL patients. We also found that expression of miR-208a-5p vs miR-1304-5p and miR-296-5p vs miR-1304-5p were positively associated in DLBCL tissue specimens. Clearly, additional investigations will be required to identify unequivocally the role of these three miRNAs in the regulation of tumor cell apoptosis and survival during DLBL-GCB tumorigenesis.

## Conclusion

The data presented have shown that miR-1304-5p, miR-208a-5p and miR-296-5p expression levels in DLBCL patients were highly correlated with their subtypes (GCB and non-GCB) and different prognoses of OS and PFS. They are promising candidates to serve as potential as biomarkers for guiding the treatment regimen of DLBCL patients.

## Supplementary Material

Supplementary Figure S1 and Tables S1-S3

## Data Availability

The datasets used and/or analyzed during the current study are available from the corresponding author on reasonable request.

## Abbreviations

ALT: alanine aminotransferase
DLBCL: diffuse large B-cell lymphoma
ECOG: Eastern Cooperative Oncology Group
FDR: false discovery rate
GCB-DLBCL: germinal center B-like DLBCL
Hb: hemoglobin
IPI: International Prognostic Index
LDH: lactate dehydrogenase
OS: overall survival
PFS: progression-free survival
qRT-PCR: quantitative reverse transcription-polymerase chain reaction
R-CHOP: rituximab, cyclophosphamide, doxorubicin, vincristine and prednisone
ROC: receiver operating characteristic
