# Supplementary material for: Differential expression of miRNAs as biomarkers for predicting the outcomes of diffuse large B-cell lymphoma patients
Source: Biosci Rep. 2021 Jun 28;41(7):BSR20201551. doi: 10.1042/BSR20201551 (PMC8239963; doi:10.1042/BSR20201551)
Supplement: Supplementary Figure S1 and Tables S1-S3 [file BSR-2020-1551_supp.pdf]

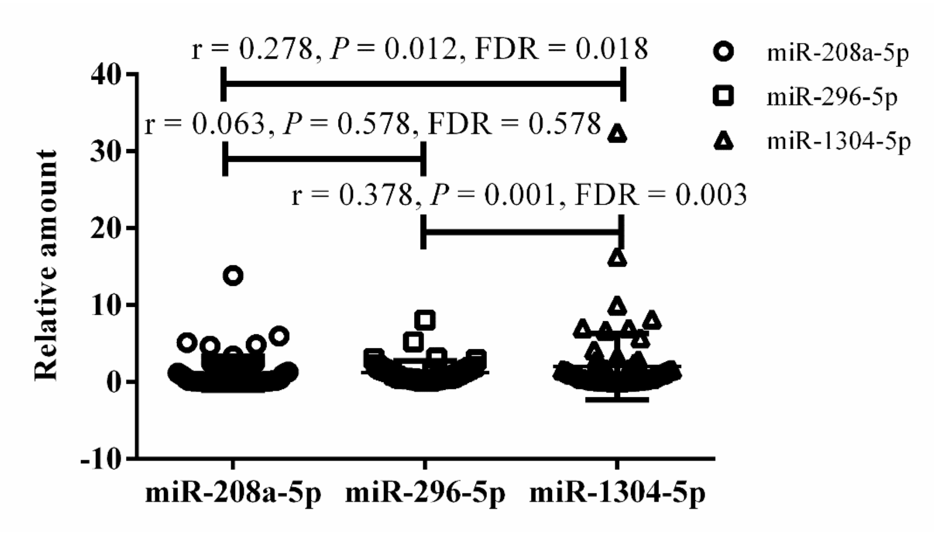

Supplementary Figure 1. Correlation analysis of miR-1304-5p and miR-208a-5p or miR-296-5p expression.

Supplementary Table 1. Primer sequences for miRNAs

miRNAs	Sequence
U6	
RT primer	5’CGCTTCACGAATTTGCGTGTCAT3’
PCR primer	Forward 5’GCTTCGGCAGCACATATACTAAAAT3’<br>Reverse 5’CGCTTCACGAATTTGCGTGTCAT3’
miR-208a-5p	
RT primer	5’GTCGTATCCAGTGCGTGTCGTGGAGTCGGCAATTGCACTGGATACGACGTATAAC3’
PCR primer	Forward 5’GAGAGCTTTTGGCCCGG3’<br>Reverse 5'GTGCGTGTCGTGGAGTCG3’
miR-296-5p	
RT primer	5’GTCGTATCCAGTGCGTGTCGTGGAGTCGGCAATTGCACTGGATACGACACAGGA3’
PCR primer	Forward 5’GAAGGGCCCCCCCTCA3’<br>Reverse 5'GTGCGTGTCGTGGAGTCG3’
miR-1304-5p	
RT primer	5’GTCGTATCCAGTGCGTGTCGTGGAGTCGGCAATTGCACTGGATACGACCACATC3’
PCR primer	Forward 5’GGGGTTTGAGGCTACAGTGA3’<br>Reverse 5'GTGCGTGTCGTGGAGTCG3’

RT, reverse transcription; PCR, polymerase chain reaction

Supplementary Table 2. Comparison of 3 differentially expressed miRNAs in DLBCL tissue specimens

**Variables**	**r**	***P*-value**	**FDR**
miR-208a-5p *vs* miR-296-5p	0.063	0.578	0.578
miR-208a-5p *vs* miR-1304-5p	0.278	0.012	0.018
miR-296-5p *vs* miR-1304-5p	0.378	0.001	0.003

The diagrams show the association between the relative expression levels of miR-208a-5p, miR-296-5p and miR-1304-5p using Pearson's correlation analysis (r, correlation coefficient). The relative expression levels of the miRNAs were calculated as $2^{-\Delta\Delta Ct}$ (= ΔCt (case) - mean ΔCt (control)).

Supplementary Table 3. Clinical characteristics of the indicated patient groups

	miR-208a-5p			miR-296-5p			miR-1304-5p		
	High (n = 40)	Low (n = 55)	*P*-value	High (n = 28)	Low (n = 67)	*P*-value	High (n = 38)	Low (n = 57)	*P*-value
**WBC (10^9/L)**	5.66 ± 2.23	5.99 ± 1.87	0.436	5.90 ± 2.29	5.83 ± 1.92	0.879	5.83 ± 1.80	5.87 ± 2.18	0.926
**PLT (10^9/L)**	215.83 ± 63.55	213.58 ± 76.57	0.880	234.89 ± 66.55	206.01 ± 71.58	0.071	222.87 ± 71.26	208.96 ± 70.96	0.353
**Hb (g/L)**	115.18 ± 15.15	122.71 ± 19.88	0.049	118.11 ± 18.59	120.13 ± 18.34	0.627	120.47 ± 20.18	118.91 ± 17.16	0.687
**ALT (IU/L)**	27.71 ± 26.07	24.18 ± 18.86	0.446	33.72 ± 33.48	22.30 ± 14.12	0.021	27.87 ± 24.65	24.19 ± 20.36	0.430
**AST (IU/L)**	25.46 ± 15.56	29.46 ± 21.83	0.325	32.99 ± 29.25	25.59 ± 13.13	0.091	27.67 ± 14.13	27.85 ± 22.43	0.965
**Cre (µmol/L)**	60.18 ± 12.24	58.35 ± 18.46	0.587	58.67 ± 16.23	59.30 ± 16.15	0.863	61.99 ± 16.18	57.20 ± 15.87	0.156

ALT, alanine aminotransferase; AST, aspartate aminotransferase; Cre, creatinine; Hb, hemoglobin; PLT, platelets; WBC, white blood cell.
